# “Last Supper” Predicts Greater Weight Loss Early in Obesity Treatment, but Not Enough to Offset Initial Gains

**DOI:** 10.3389/fpsyg.2018.01335

**Published:** 2018-08-02

**Authors:** Jena Shaw Tronieri, Thomas A. Wadden, Nasreen Alfaris, Ariana M. Chao, Naji Alamuddin, Robert I. Berkowitz, Rebecca L. Pearl

**Affiliations:** ^1^Department of Psychiatry, Perelman School of Medicine, University of Pennsylvania, Philadelphia, PA, United States; ^2^Obesity, Endocrine, and Metabolism Center, King Fahad Medical City, Riyadh, Saudi Arabia; ^3^Department of Biobehavioral Health Sciences, School of Nursing, University of Pennsylvania, Philadelphia, PA, United States; ^4^Department of Medicine, Perelman School of Medicine, University of Pennsylvania, Philadelphia, PA, United States; ^5^Department of Child and Adolescent Psychiatry and Behavioral Sciences, Children’s Hospital of Philadelphia, Philadelphia, PA, United States; ^6^Department of Surgery, Perelman School of Medicine, University of Pennsylvania, Philadelphia, PA, United States

**Keywords:** obesity, weight loss, pre-treatment weight change, weight loss predictors, weight fluctuation, behavior therapy

## Abstract

**Background:** Many participants experience clinically significant fluctuations in weight before beginning a behavioral weight loss program. Pre-treatment weight gain, often referred to as the “last supper” effect, may limit total weight loss from the time of the pre-treatment screening visit and could be an indicator that a participant will respond poorly to behavioral intervention.

**Methods:** Data were from the weight loss phase of a two-phase weight loss maintenance trial, in which 178 participants with obesity (screening BMI = 40.5 ± 6.0 kg/m^2^, 87.6% female; 71.3% black) were provided with a 14 week lifestyle intervention that included a meal replacement diet. Participants were categorized as having gained >1.15%, remained weight stable, or lost >1.15% of initial weight between the pre-treatment screening visit and the first treatment session (48.7 ± 29.4 days). We first examined whether the weight change groups differed in baseline eating characteristics (e.g., emotional eating, self-regulation, craving frequency) using one-way ANCOVAs. Linear mixed models were then used to compare weight change groups on total weight loss from the screening visit to week 14 and in-treatment weight loss from weeks 1 to 14.

**Results:** Nearly half of the sample (48.9%) gained >1.15% of initial weight during the pre-treatment period (+2.5 ± 1.2%); 41.0% remained weight stable (+0.2 ± 0.6%); and 10.1% lost >1.15% of initial weight (-2.2 ± 0.9%). There were no significant differences between the groups in baseline eating characteristics. As measured from the screening weight, the weight-gain group had a lower total loss of 6.8%, compared to 7.8% in the weight stable group (*p* = 0.02) and 9.0% in the weight-loss group (*p* = 0.003). The weight-gain group lost more weight in the first 4 weeks of treatment, but in-treatment losses did not differ among the groups at week 14.

**Conclusion:** Pre-treatment weight gain was not an indicator of a poor response to a behavioral weight loss intervention and was associated with greater weight loss early in treatment. However, weight gain during the pre-treatment period may limit the total weight loss that participants achieve from the time that they first enroll in a weight loss program.

## Introduction

Before joining a behavioral weight loss program, potential participants typically attend one or more pre-treatment screening visits in which staff assess their eligibility, obtain informed consent, and measure their pre-treatment characteristics (e.g., Ryan et al., 2003). In many studies, individuals who enroll are then asked to wait while a cohort of participants is recruited to fill spaces in a group or to allow researchers to randomize participants simultaneously. Prior studies of the period between participants’ initial screening and the start of treatment have reported average delays of 42 and 50 days (West et al., 2011; Kerrigan et al., 2016, respectively). A substantial minority of participants experience clinically significant weight changes during this time, with 17–23% losing more than 1.15% of initial weight, and 16–30% gaining over 1.15% (West et al., 2011; Kerrigan et al., 2016). Pre-treatment weight gain is sometimes referred to as the “last supper" effect, suggesting that some individuals indulge in overeating when anticipating a period of dietary restriction.

Two studies have investigated whether pre-treatment weight change predicts later weight loss during behavioral treatment programs. West et al. (2011) found that participants who lost >1.15% of initial weight prior to beginning treatment also lost more weight between week 1 of treatment and the 6 month assessment than participants who had gained >1.15% or remained weight stable within 1.15% of their screening weight. However, the authors also noted that individuals who had gained weight before treatment had a disproportionately lower representation in the most effective treatment group. It was therefore possible that chance differences in treatment assignment had produced the observed differences in post-treatment weight loss.

In a second study, Kerrigan et al. (2016) found that individuals who lost weight before beginning treatment had lower levels of baseline hedonic hunger, uncontrolled eating, and emotional eating, and higher levels of weight-related self-efficacy. In their study, weight losses between week 1 of treatment and month 6 did not differ among the pre-treatment weight change groups. However, total weight losses between the screening visit and month 6 were largest for individuals who had lost weight before treatment. The authors reported that pre-treatment weight change category did not interact with treatment condition in predicting weight loss, but they did not describe whether participants in each weight change group were evenly distributed between their two treatment conditions.

Data from the weight loss phase of a two-phase study in which all participants were initially enrolled in the same 14 weeks, group lifestyle intervention (Tronieri et al., 2017) provided a unique opportunity to investigate the role of pre-treatment weight change without the potential confounding effects of treatment assignment. Like previous studies, we first categorized participants as either losing >1.15% of initial weight, remaining stable within 1.15%, or gaining >1.15% of initial weight between their screening visit and the start of the treatment program. We then examined whether pre-treatment weight change groups differed in weight loss during treatment (i.e., from weeks 1 to 14) or in total loss from the initial screening visit (i.e., from the screening visit to week 14). We also attempted to replicate Kerrigan et al.’s (2016) examination of differences between the pre-treatment weight change groups in baseline eating characteristics.

## Materials and Methods

### Participants

Adults with obesity were recruited to participate in a two-phase study that consisted of a 14 week non-randomized group lifestyle intervention, followed by a 52 week randomized controlled trial (RCT) that assessed weight loss maintenance with lorcaserin versus placebo, both combined with behavioral weight loss maintenance counseling. The study’s design, methods, and inclusion/exclusion criteria (Tronieri et al., 2017), as well as the primary results of the 52 week RCT (Tronieri et al., 2018), have been reported previously. Only data from the 14 week non-randomized lifestyle intervention were used in the present study.

Eligible participants were aged 21–65 years, had a body mass index (BMI) ≥33 kg/m^2^ and ≤55 kg/m^2^ (or ≥30 kg/m^2^ with an obesity-related comorbidity), and had no serious medical or psychological conditions (e.g., diabetes mellitus, recent cardiovascular disease, current major depressive disorder). This study was carried out in accordance with the requirements of the Institutional Review Board at the University of Pennsylvania, which approved the study protocol. All participants gave written informed consent in accordance with the Declaration of Helsinki.

### Procedures

#### Screening and Enrollment

Participants were recruited in three cohorts that were screened from January 16 to April 1, 2015, June 3 to August 29, 2015, and October 12, 2015 to January 21, 2016. Interested individuals were pre-screened by telephone to assess preliminary eligibility and interest in the study. Those who appeared eligible then completed an in-person screening visit that included: a behavioral evaluation conducted by a psychologist; obtaining informed consent; and assessing medical eligibility (Tronieri et al., 2017). Individuals who remained eligible also completed an electrocardiogram (EKG), urine pregnancy test (for females of child-bearing age), and fasting blood draw to determine final eligibility criteria. Baseline questionnaires were completed at home after the screening visit and prior to the first treatment session.

#### Weight Loss Intervention

Participants were provided with 14 weekly, 90-minute group lifestyle modification sessions (10–15 participants per group). The goal of this intervention was to help participants lose at least 5% of initial weight in order to qualify for the weight loss maintenance RCT. Participants were prescribed a 1,000–1,200 kcal per day meal-replacement diet that included four servings of a liquid shake (Health Management Resources, HMR; 160 kcal/shake), a prepackaged entrée (250–300 kcal), 1–2 servings of fruit, and a salad or vegetable serving. The use of shakes was terminated gradually between weeks 12 and 14. Participants were also instructed to gradually increase their physical activity and to self-monitor their food intake (including calories), weight, and daily activity.

### Measures

Body weight was measured at the screening visit and at all treatment visits using a digital scale (Tanita BWB-800). Participants were dressed in light clothing, without shoes. As in previous studies (West et al., 2011; Kerrigan et al., 2016), percent weight change between the screening visit and first treatment session was used to categorize participants as having either gained more than 1.15% (weight-gain group), remained stable within 1.15% (weight stable group), or lost more than 1.15% (weight-loss group) relative to their screening visit weight. As described by West et al. (2011), the 1.15% cutoff was selected to represent a clinically significant weight change of approximately half of the amount frequently used to define weight maintenance over a longer period (e.g., 2.3 kg over 6 months).

Baseline questionnaires that assessed emotional eating, self-regulation (cognitive restraint and uncontrolled eating), and craving frequency were used to compare the baseline characteristics of the pre-treatment weight change groups. The Eating Inventory (EI; Stunkard and Messick, 1988) is a commonly used questionnaire that measures several eating traits. The present study applied the 18-item scoring, which assesses uncontrolled eating, cognitive restraint, and emotional eating, to match analyses conducted by Kerrigan et al. (2016). This revised scoring system has shown improved factor loading and adequate reliability and validity (Karlsson et al., 2000). We also included the Food Craving Inventory (FCI; White et al., 2002), which measures frequency of cravings for specific foods over the past 28 days. The total score of the FCI, which has also shown high reliability and validity, was used in the present analyses.

### Statistical Analyses

Pre-treatment weight change groups were first compared on demographic characteristics using chi square tests and one-way ANOVAs, and Tukey’s *post hoc* tests were used to evaluate between-group differences. We then compared the baseline eating traits (e.g., emotional eating, self-regulation, and craving frequency) of the pre-treatment weight change groups using one-way ANCOVAs (controlling for baseline differences).

We used linear mixed models with residual maximum likelihood to determine whether the groups differed in their weight change during treatment (i.e., from weeks 1 to 14) or in their total weight change from the screening visit to week 14. Unconditional models were used to determine the appropriate model shape (e.g., linear, quadratic, piece-wise with breakpoints tested at weeks 2–7) and variance-covariance structure based on model fit criteria (e.g., AIC, -2 log likelihood) (Gallop and Tasca, 2009). Estimated weight losses from the start of treatment and from the screening visit were calculated and compared between pre-treatment weight change groups using least squared means.

We conducted an exploratory follow-up analysis to determine whether the relationship between pre-treatment weight change (measured continuously) and weight loss in treatment (i.e., from weeks 1 to 14) was mediated by early weight change (from weeks 1 to 5, as identified in the primary analysis). The indirect effect (*ab* path) was computed to measure the change in the relationship between pre-treatment weight change and weight loss in treatment when controlling for early weight loss (*c* path – *c*′ path). This mediation effect was estimated by first regressing early weight change on pre-treatment weight change (*a* path), and subsequently regressing in-treatment weight loss on early weight change while controlling for pre-treatment weight change (*b* path) (Preacher and Hayes, 2008). Bootstrapping was conducted using the PROCESS script for SPSS (Hayes, 2017) with 5,000 resamples, and 95% bias-corrected confidence intervals were computed to determine the statistical significance of the indirect effect.

## Results

Participants (*N* = 178) had a mean (±SD) age of 44.2 ± 11.2 years; 87.6% were female and 71.3% were black (21.9% white). Their mean BMI at the screening visit was 40.5 ± 6.0 kg/m^2^. Participants waited an average of 48.7 ± 29.4 days (range 8–159 days) between the screening visit and first treatment session. The average participant gained 1.1 ± 1.8% of initial weight (1.2 ± 2.1 kg) during this time. Only 10.1% of participants (*n* = 18) lost >1.15% of initial weight between the screening and first treatment session, with average losses among this group of -2.2 ± 0.9% (range -4.6 to -1.2%). Weight remained within 1.15% of the screening weight for 41.0% of the sample (*n* = 68; mean weight change of +0.2 ± 0.6%; range -1.1 to +1.1%). Nearly half of the sample (48.9%, *n* = 83) gained >1.15% of initial weight, with average gains of 2.5 ± 1.2% (range: +1.2 to +6.6%).

We observed that weight gain related to the winter holidays could have affected the pre-treatment weight change of individuals in our third participant cohort (recruitment from October 12, 2015 to January 21, 2016, *n* = 55). Of the 35 participants recruited prior to January 1, 77.1% (*n* = 27) gained weight before beginning treatment, compared to 42.0% of the remainder of the sample (*n* = 60 of 143). Of the participants recruited before the holidays, 17.1% (*n* = 6) remained weight stable and 5.7% lost weight (*n* = 2), compared to 46.9% (*n* = 67 of 143) and 11.2% (*n* = 16 of 143) of participants not recruited during that period. After controlling for days between the screening and first treatment visit, individuals recruited before the holidays had 3.6 times greater odds of gaining weight than those recruited at other times (95% CI: 1.22–10.67, *p* = 0.02).

### Characteristics of the Pre-treatment Weight Change Groups

There were no significant differences between the pre-treatment weight change groups in any demographic characteristic (**Table 1**). Participants who remained weight stable waited for fewer days between the screening and first treatment session (39.4 ± 24.6 days) than those who gained weight (55.4 ± 31.5 days, *p* = 0.001). The difference between weight-loss and weight stable participants (53.6 ± 27.8 days) was not statistically significant (*p* = 0.14). The weight-loss group had a higher mean BMI at screening than the weight-gain group (43.8 ± 7.2 vs. 39.5 ± 6.0 kg/m^2^, *p* = 0.01). The weight stable group (40.9 ± 5.3 kg/m^2^) did not differ significantly from either the weight-loss (*p* = 0.18) or weight-gain group (*p* = 0.20) in screening visit BMI. We controlled for BMI at screening and the number of days between the screening and first treatment visit as covariates in all subsequent analyses.

**Table 1:** Differences among pre-treatment weight change categories in demographic characteristics and baseline psychosocial variables.

	Loss (*n* = 18)	Stable (*n* = 73)	Gain (*n* = 87)	*p*
Age	43.0 (2.7)	43.9 (1.3)	44.7 (1.2)	0.81
Sex (female), *n* (%)	16 (88.9%)	65 (89.0%)	75 (86.2%)	0.86
Race (black), *n* (%)	12 (66.7%)	55 (75.3%)	60 (69.0%)	0.83
Screening BMI	43.8 (1.4)	41.0 (0.7)	39.4 (0.6)	0.01^a^
Week 1 BMI	42.9 (1.4)	41.0 (0.7)	40.5 (0.6)	0.29
Days between screening visit and week 1	53.6 (6.7)	39.4 (3.3)	55.4 (3.1)	0.002^b^
Number of sessions attended (out of 14)	10.7 (0.8)	12.4 (0.4)	11.9 (0.3)	0.12
Attrition, *n* (%)	5 (27.8%)	10 (13.7%)	14 (16.1%)	0.28
Uncontrolled eating (EI)	28.3 (6.7)	30.1 (3.5)	30.0 (3.1)	0.97
Emotional eating (EI)	32.6 (9.8)	53.2 (5.1)	48.0 (4.5)	0.18
Cognitive restraint (EI)	51.6 (5.9)	44.4 (3.1)	41.4 (2.8)	0.28
Food cravings (FCI)	2.2 (0.2)	2.3 (0.1)	2.3 (0.1)	0.99


*Values are means and standard errors, except as otherwise noted. Values for eating traits and session attendance were adjusted for screening visit BMI and number of days between screening and week 1 of treatment. ^*a*^Post hoc (Tukey) comparisons indicated a significant difference between the gain and loss groups (p = 0.01). ^*b*^Post hoc (Tukey) comparisons indicated a significant difference between the weight-gain and weight stable groups (p = 0.001). No other significant differences were observed.*

There were no significant differences between the weight change groups in any baseline eating characteristic (**Table 1**). Neither session attendance nor attrition (defined as dropout prior to week 12) differed significantly by pre-treatment weight change category. Screening BMI and days between the screening and first treatment session did not significantly predict any of the behavioral outcomes.

### Total Weight Loss From the Screening Visit to Week 14

A piecewise model with a breakpoint at week 5 best fit the data, indicating a change in the average rate of weight loss after 4 weeks of treatment. Screening BMI and days between the screening and first treatment session did not significantly predict weight change in treatment.

**FIGURE 1:**
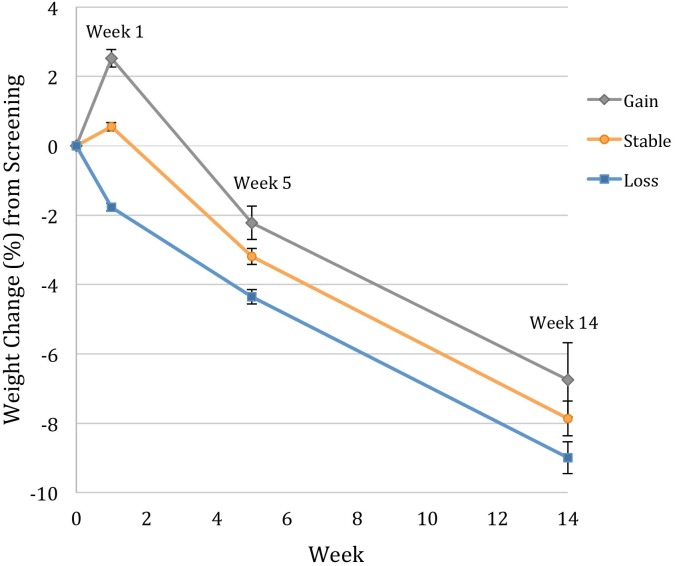
Modeled weight change trajectories of participants who gained >1.15%, remained weight stable within 1.15%, and lost >1.15% between the screening visit and week 1 of treatment. Data for percentage weight change from the screening visit (week 0) are estimated marginal means (±SE) for the intention-to-treat population (*N* = 178), controlling for screening visit BMI and number of days between screening and week 1 of treatment. Individuals who gained weight before treatment lost significantly more weight between weeks 1 and 5 of treatment, but maintained lower total losses from the screening visit at both weeks 5 and 14.

**FIGURE 2:**
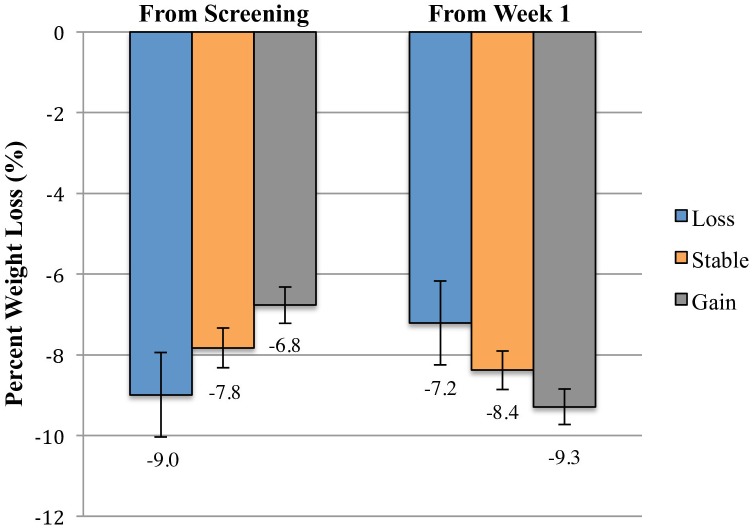
Mean percent weight losses between the screening visit and week 14 and between weeks 1 and 14 of participants who gained >1.15%, remained weight stable within 1.15%, and lost >1.15% between the screening visit and week 1 of treatment. Data are estimated marginal means (±SE) at week 14 for the intention-to-treat population (*N* = 178), controlling for screening visit BMI and number of days between screening and week 1 of treatment. The weight-gain group had lower total percent losses from the screening visit than the weight stable group (*p* = 0.02) and the weight-loss group (*p* = 0.003). The difference between the weight-loss and weight-gain groups in weight loss from weeks 1 to 14 did not reach statistical significance (*p* = 0.07). No other groups differed significantly at either time point.

**Figure 1** illustrates the weight change trajectories of each pre-treatment weight change group between the screening visit and week 14 of treatment. Participants who had gained weight prior to treatment lost weight faster between weeks 1 and 5 than those who had remained weight stable (*p* = 0.001) or who lost weight (*p* < 0.001), with losses of 4.7, 3.7, and 2.9%, respectively, during that period. However, relative to their screening weight, the weight-gain group still had lower total losses at week 5 of only 2.2%, compared to 3.2% for the weight stable group (*p* = 0.002) and 4.6% for the weight-loss group (*p* < 0.001). The weight-loss group also differed significantly from the weight stable group (*p* = 0.007; see **Table 2**). From weeks 5 to 14, the groups did not differ in rate of weight loss (*p* = 0.94), losing an additional 4.6% (*SE* = 0.3), 4.6% (*SE* = 0.3), and 4.3% (*SE* = 0.7), respectively. The weight-gain group therefore continued to have lower total losses relative to their screening weight of 6.8% (95% CI: 5.8–7.6%), compared to 7.8% for the stable group (*p* = 0.02, 95% CI: 6.9–8.8%), and 9.0% for the loss group (*p* = 0.003, 95% CI: 6.9–11.1%; loss vs. stable *p* = 0.10; **Figure 2**).

**Table 2:** Differences among pre-treatment weight change groups in weight change percentage, controlling for screening visit BMI and number of days between screening and week 1 of treatment.

	Loss (*n* = 18)	Stable (*n* = 73)	Gain (*n* = 87)
Pre-treatment weight change (screening to week 1)	-1.8% (0.2)^a^	+0.5% (0.1)^b^	+2.5% (0.1)^c^
Early treatment weight change (weeks 1 to 5)	-2.9% (0.5)^a^	-3.7% (0.2)^a^	-4.7% (0.2)^b^
In-treatment weight change^∗^ (weeks 1 to 14)	-7.2% (1.0)	-8.4% (0.5)	-9.3% (0.4)
Total weight change at week 5 (screening to week 5)	-4.6% (0.5)^a^	-3.2% (0.2)^b^	-2.2% (0.2)^c^
Total weight change (screening to week 14)	-9.0% (1.1)^a^	-7.8% (0.5)^a^	-6.8% (0.5)^b^


*Values are estimated marginal means, adjusted for screening visit BMI and number of days between screening and week 1 of treatment. Superscripts indicate significant differences (p < 0.05) for each contrast based on least squared mean differences.*^∗^*The difference between loss and gain groups in in-treatment weight change did not reach statistical significance (p = 0.07).*

### In-treatment Weight Loss From Weeks 1 to 14

**FIGURE 3:**
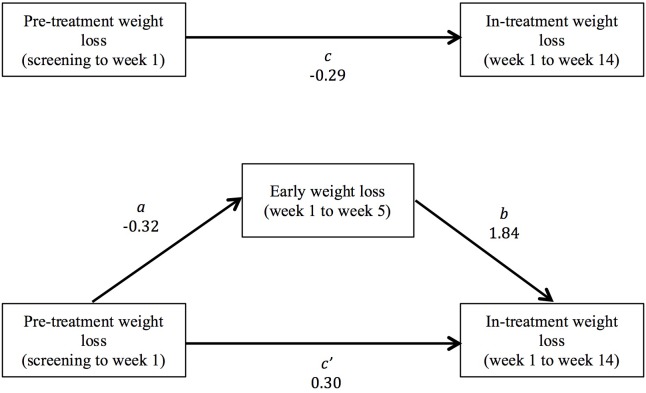
Early weight loss from weeks 1 to 5 mediated the relationship between pre-treatment weight change and weight loss in treatment (from weeks 1 to 14). The bivariate total relationship between pre-treatment weight change and total weight loss form weeks 1 to 14 was negative and not statistically significant (*c* path). The direct relationship between pre-treatment weight change and weight loss in treatment, controlling for early weight loss, was positive (*c*′ path). However, pre-treatment weight change was also negatively associated with early weight loss (*a* path), and early weight loss was a positive predictor of total in-treatment weight loss (*b* path). Therefore, the indirect effect of pre-treatment weight change on total weight loss was negative (*ab* path).

The pre-treatment weight change groups did not differ significantly in weight loss between weeks 1 and 14 (**Table 2**). Early weight loss from weeks 1 to 5 mediated the relationship between pre-treatment weight change and weight loss during treatment (**Figure 3**). Prior to including the mediator variable (early weight loss) in the analysis, the relationship between pre-treatment weight change and weight loss in treatment from weeks 1 to 14 was negative and not statistically significant (*c* path*; b* = -0.29, *SE* = 0.17, 95% CI: -0.63–0.06). After adding early weight loss to the analysis, greater pre-treatment weight loss was directly associated with greater weight loss in treatment from weeks 1 to 14 (*c*′ path*; b* = 0.30, *SE* = 0.11, 95% CI: 0.08–0.52). However, the indirect effect of pre-treatment weight change on in-treatment weight loss was negative (*ab* path; *b* = -0.59, *SE* = 0.15, 95% CI: -0.88 to -0.31). Greater pre-treatment weight loss predicted smaller early weight loss from weeks 1 to 5 (*a* path; *b* = -0.32, *SE* = 0.08, 95% CI: -0.47 to -0.17), and the amount of early weight loss was positively associated with in-treatment loss from weeks 1 to 14 (*b* path; *b* = 1.84, *SE* = 0.11, 95% CI: 1.63–2.05).

## Discussion

This study provided further evidence that many individuals with obesity experience significant fluctuations in weight before beginning a behavioral weight loss program. Nearly half of participants awaiting the start of a group treatment program gained a clinically meaningful amount (>1.15% of their screening weight), with average gains of 2.5% among this group. This “last supper effect” was associated with early losses within the first 4 weeks of treatment that were 1.0% larger than those of individuals who had remained weight stable, and 1.8% larger than participants who had lost weight prior to beginning the treatment. This differential initial rate of weight loss could represent a regression to the mean (i.e., a return to screening weight). However, the magnitude of the early loss was not large enough to fully reverse the pre-treatment weight gain. The weight-gain group had lower total losses relative to their screening weights after 14 weeks of treatment, losing 1.0% less than the weight stable group and 2.2% less than the weight-loss group. The minority of the sample who lost a clinically meaningful amount of weight before beginning treatment did not differ significantly from weight stable participants in either early or total weight loss in treatment.

The association between pre-treatment weight change and weight change in treatment was mediated by early weight loss during the first 4 weeks of treatment. The bivariate relationship between pre-treatment weight change and total weight loss form weeks 1 to 14 was negative and not statistically significant. However, a suppression effect was present (MacKinnon et al., 2000). The direct effect of pre-treatment weight change on in-treatment weight loss was positive. This indicated that when early weight loss was held constant, larger pre-treatment losses were associated with larger in-treatment weight losses. However, the indirect effect of pre-treatment weight change via early weight loss was negative. Larger pre-treatment losses were associated with smaller early weight losses, and smaller early losses predicted smaller losses in treatment. A positive direct relationship and negative mediated relationship tend to cancel each other out when the mediator variable is not included (MacKinnon et al., 2000). This suppression effect may explain why the study by Kerrigan et al. (2016), which did not consider the role of early weight loss, also failed to find a bivariate association between pre-treatment and in-treatment weight change.

In **Table 3** we present the primary outcomes of the present study alongside those of previous studies by West et al. (2011) and Kerrigan et al. (2016). In comparison to these studies, a larger percentage of participants in the present sample gained weight prior to beginning treatment, and a smaller percentage lost weight. Although exploratory analyses suggested that the holiday timing of one cohort was associated with a greater likelihood of pre-treatment weight gain, pre-treatment weight gain remained more common (42%), and weight loss less common (11%), in our sample when these participants were excluded. Although the average number of days between the screening and start of treatment was similar among the three studies, our study was unique in finding that individuals who remained weight stable waited less time than those who gained weight. These findings may suggest that our sample had a greater tendency toward weight fluctuation when treatment was delayed. It may be that demographic differences contributed to these results. In comparison to previous samples (West et al., 2011; Kerrigan et al., 2016, respectively), our sample appears to have had higher BMIs (mean of 40.5 kg/m^2^ compared to 35.6 and 35.2 kg/m^2^) and was more racially diverse (71.3% black compared to 27.4% black and 34.2% non-white).

**Table 3:** Results of three studies examining differences between pre-treatment weight change groups in weight change during a behavioral weight loss program.

	Present study			West et al., 2011			Kerrigan et al., 2016		
			
	Loss	Stable	Gain	Loss	Stable	Gain	Loss	Stable	Gain
	(*n* = 18)	(*n* = 73)	(*n* = 87)	(*n* = 110)	(*n* = 292)	(*n* = 78)	(*n* = 47)	(*n* = 152)	(*n* = 84)
Proportion of sample (%)	10	41	49	23	61	16	17	54	30
Days between screening visit and week 1	53.6^a,b^	39.4^a^	55.4^b^	49.3	48.6	53.6	45.2	41.5	41.4
Pre-treatment weight change (screening to week 1)	-1.8%^a^	+0.5%^b^	+2.5%^c^	-2.4 kg^a^	+0.1 kg^b^	+2.1 kg^c^	-2.2%^a^	+0.1%^b^	+2.4%^c^
In-treatment weight change (from week 1)	-7.2%	-8.4%	-9.3%	-8.9 kg^a^	-6.1 kg^b^	-5.7 kg^b^	-9.2%	-9.6%	-9.8%
Total weight change (from screening)	-9.0%^a^	-7.8%^a^	-6.8%^b^	~11.3 kg	~6.0 kg	~3.6 kg	-11.2%^a^	-9.4%^b^	-7.7%^c^


*Superscripts indicate significant differences (p < 0.05) for each contrast. Values for total weight change in West et al. (2011) were not described by the authors and were therefore estimated by combining pre-treatment and in-treatment weight change.*

The results of these three studies consistently support the conclusion that the “last supper effect” is associated with lower total weight loss between the screening visit and the end of treatment. Because greater weight loss is associated with larger improvements in cardiometabolic risk factors (Wing et al., 2011), it is possible that the total physiological benefits of participation in a weight loss program are attenuated for individuals who gain weight before the start of treatment. It also suggests that cardiometabolic outcomes should be measured close to the start of treatment, rather than at the screening visit, to most accurately reflect the effects of the treatment condition.

West et al. (2011) found that weight-gain participants also lost less in treatment than weight-loss participants, while Kerrigan et al. (2016) observed slightly larger losses among weight-gain participants that did not differ significantly from the other groups (*p* = 0.13). As described above, a non-uniform distribution of the weight change groups among different treatment conditions could have affected these studies’ results, particularly for West et al. (2011). In the present study, all participants enrolled in the same 14 week behavioral program. By modeling weight change longitudinally, we demonstrated that weight loss in the first 4 weeks of treatment was largest for the weight-gain group. Similar to Kerrigan et al. (2016), the weight-gain group had marginally greater weight losses at the end of treatment, but this difference was not statistically significant. The results of these latter two studies do not support the idea that pre-treatment weight gain predicts poor weight loss in behavioral treatment.

The results of these three behavioral studies parallel the more extensively researched link between pre-treatment weight change and post-surgical weight loss in bariatric surgery. Analyses that have statistically combined study results (Kadeli et al., 2012; Livhits et al., 2012) suggest that weight loss before surgery results in greater total losses from the start of the pre-surgical program, but does not impact post-surgical weight loss. It would be useful to examine whether individuals who engage in self-directed dieting also experience weight fluctuations between forming the intention to lose weight and beginning to make dietary changes.

Previous studies have hypothesized that individual patient characteristics might affect pre-treatment weight stability. Kerrigan et al. (2016) found that individuals who lost weight before treatment had lower emotional eating and uncontrolled eating as measured by the EI, and lower hedonic eating as measured by the Power of Food Scale (PFS). We were not able to replicate these differences in our sample. It is possible that the smaller sample size in our study limited our ability to detect these differences. The winter holidays may also have been an alternative source of weight gain for a subset of our sample. Neither of the two previous studies reported on time frame of recruitment, so we cannot determine if holiday weight gain influenced their results. The differences in screening BMI between the loss and gain groups could indicate that the pre-treatment weight change itself represented a regression to the mean in the present study. However, screening BMI predicted only a small percentage of the variance (5.0%) in pre-treatment weight change, suggesting that other factors contributed to this phenomenon. Additionally, the results of our primary analyses were consistent with those of previous studies that did not observe differences in screening BMI among the weight change categories. However, further replication is needed to determine whether the affect of pre-treatment weight change on weight loss is distinct from that of screening weight.

The present study contributes to the growing literature on the effect of pre-treatment weight changes on weight loss during obesity interventions. A significant strength of this study was the evaluation of the effect of pre-treatment weight change without the potential confounding effects of treatment assignment. However, the relatively small sample size may have limited our ability to detect differences between pre-treatment weight change groups, particularly in light of the small percentage of the sample who lost weight before beginning treatment. Additionally, only 14 weeks of treatment were provided during the behavioral weight loss phase of this study, so we were not able to evaluate the effects of pre-treatment weight change on longer-term weight loss outcomes.

Because this study was based on a secondary analysis of existing data, we may not have captured important differences among the pre-treatment weight change groups. For example, the timing of completion of the eating behavior questionnaires may have affected our ability to detect differences among the groups. It would be useful to measure participants’ eating behaviors at both screening and week 1 of treatment to best determine the relationship between these behaviors and pre-treatment weight change. We also do not know if the groups would have differed in weight stability outside of the pre-treatment period. Due to the nature of obtaining pre-treatment weight change data during recruitment for behavioral weight loss programs, no study has been able to evaluate whether participants’ weight change fluctuations are a product of anticipating participation in a weight loss program (the “last supper effect” and self-initiated weight loss), or would have occurred regardless of whether the individual had decided to lose weight. A study in which participants are randomized to either begin a weight loss program or to not attempt weight loss would be needed to address this question.

The present study found further evidence that many individuals experience clinically meaningful weight changes during the period between the initial screening visit and start of a behavioral weight loss program. Weight gain during this period resulted in larger early weight losses, but smaller total losses from the time of the screening visit. Individuals interested in participating in a behavioral weight loss program should be cautioned that weight gain while waiting to start treatment can detract from the overall benefit of their participation. However, pre-treatment weight gain did not affect total weight loss during the treatment, suggesting that participants who gain weight after their screening visit will still respond appropriately to the intervention and should not be excluded from clinical trials.

## Data Availability

### Restrictions Apply to the Datasets

The raw data supporting the conclusions of this manuscript will be made available by the authors, without undue reservation, to any qualified researcher for the purpose of replicating the analyses reported in the present manuscript. The datasets for this manuscript are not publicly available because the research team has not yet completed initial data analyses. Requests to access the datasets should be directed to Thomas A. Wadden, Ph.D. (wadden@pennmedicine.upenn.edu).

## Author Contributions

TW was responsible for the conception and design of the parent randomized controlled trial and co-wrote the study protocol with NasA. JT and RP provided behavioral weight loss treatment, and RB, NasA, NajA, and AC provided medical monitoring to patients during the randomized trial. JT organized the database, performed the statistical analysis, and wrote the first draft of the present manuscript. All authors contributed to manuscript revision, read and approved the submitted version.

## Conflict of Interest Statement

JT and NajA disclose serving as consultants for Novo Nordisk. TW reports serving on advisory boards for Novo Nordisk and Weight Watchers and has received grant support on behalf of the University of Pennsylvania from Eisai Inc. and Novo Nordisk. TW and AC have received grant support on behalf of the University of Pennsylvania from Shire Pharmaceuticals. RB has received consulting fees from Eisai Inc. RP discloses serving as a consultant for Weight Watchers. The remaining author declares that the research was conducted in the absence of any commercial or financial relationships that could be construed as a potential conflict of interest.
